# Highlighting the Role of Prenatally Administered Drugs in the Production of Dental Enamel Defects in Rats by Polarized Light Microscopy

**DOI:** 10.3390/biomedicines13030575

**Published:** 2025-02-25

**Authors:** Mihai Popescu, Marilena Bătăiosu, Stelian-Mihai-Sever Petrescu, Mihaela Ionescu, Marius Ciprian Văruț, Diana Elena Vlăduțu, Tiberius-Cătălin Dudan, Adina-Monica Chiriac, Camelia Fiera (Maglaviceanu), Veronica Mercuț

**Affiliations:** 1 Department of Pedodontics, Faculty of Dental Medicine, University of Medicine and Pharmacy of Craiova, 200349 Craiova, Romania; mihai_15005@yahoo.com (M.P.); marilena.bataiosu@yahoo.com (M.B.); dalmmiy@yahoo.com (A.-M.C.); cameliamaglaviceanu@yahoo.com (C.F.); 2 Department of Orthodontics, Faculty of Dental Medicine, University of Medicine and Pharmacy of Craiova, 200349 Craiova, Romania; 3 Department of Medical Informatics and Biostatistics, Faculty of Dental Medicine, University of Medicine and Pharmacy of Craiova, 200349 Craiova, Romania; 4 Department of Biophysics, University of Medicine and Pharmacy of Craiova, 200349 Craiova, Romania; varutmarius@yahoo.com; 5 Department of Prosthodontics, University of Medicine and Pharmacy of Craiova, 200349 Craiova, Romania; diana.vladutu@umfcv.ro (D.E.V.); veronica.mercut@umfcv.ro (V.M.); 6 Department of Oral and Maxillo-Facial Surgery, University of Medicine and Pharmacy of Craiova, 200349 Craiova, Romania; tiberiusdudan@gmail.com

**Keywords:** developmental defects of enamel, drugs, polarized light microscopy, statistical analysis, Wistar rats

## Abstract

**Background/Objectives:** Although factors acting both prenatally and postnatally are taken into consideration, the etiopathogenesis of developmental defects of enamel (DDE) is not fully understood. Among the medications used for a variety of ailments, amoxicillin and cefaclor are indicated as having a part in the development of DDE. The objective of the present study was to reproduce DDE in the laboratory in rats by administering amoxicillin, ibuprofen, and cefaclor. These lesions were subsequently diagnosed using polarized light microscopy (PLM). **Methods:** This study was conducted on Wistar rats, which were given prenatally drugs possibly involved in the production of DDE. After macroscopic examination and identification of enamel defects, bright-field microscopy (BFM) and PLM examination were performed. **Results:** The group that received cefaclor was the most affected, according to the data gathered from this study. This group was followed by the groups that received amoxicillin in a double dose, ibuprofen, amoxicillin in a standard dose, and the control group. **Conclusions:** In the control group, DDE was identified in a reduced number, resulting in the fact that there are other factors involved, besides the drugs administered, in the development of DDE. Following this research, it was concluded that DDE in the form of demineralization was more frequently recorded in the cefaclor and ibuprofen groups, while DDE in the form of hypoplasia was more frequently recorded in the double-dose and standard-dose amoxicillin groups.

## 1. Introduction

The etiopathogenesis of developmental defects of dental enamel (DDE) is incompletely elucidated, and factors acting pre- and postnatally are considered. Prenatal factors are represented by socio-economic status, nutritional deficiencies, various affections or infections of the mother, multiple births, low birth weight, and prematurity [1,2].

The postnatal factors acting during the period of odontogenesis, with a role in the development of DDE, are represented by nutritional deficiencies, systemic conditions, and local trauma [3]. Among the conditions associated with fever, varicella, measles, pneumonia, frequent episodes of diarrhea, asthma, renal failure, rubella, parotitis, tonsillitis, and adenoid infections were specifically mentioned [4,5,6].

Among the drugs used in these conditions, amoxicillin and cefaclor are mentioned as having a part in the development of DDE [7,8,9]. DDE and amoxicillin, ibuprofen, and cefaclor given during the first few years of a child’s life were found to be significantly correlated in a prior statistical analysis [10]. Because some animals’ odontogenesis is similar to that of humans, studies conducted on laboratory animals enable the investigation of secondary pharmacological effects.

The objective of the present study was to reproduce DDE in the laboratory in rats by administering amoxicillin, ibuprofen, and cefaclor. These lesions were subsequently diagnosed using polarized light microscopy (PLM).

## 2. Materials and Methods

The present study was approved by the Ethics Committee of the University of Medicine and Pharmacy of Craiova, Romania (approval reference no. 7/20 January 2021). The study was conducted on Wistar rats, which were given prenatally drugs possibly involved in the production of DDE. In order to achieve the proposed objectives, 5 batches of 3 adult female Wistar rats were constituted.

The first group was represented by rats in the control group, which were not given drugs. The second group was represented by rats that received 50 mg/kg amoxicillin (the standard dose recommended for children). The third group was represented by rats given 100 mg/kg amoxicillin (double dose compared to the standard dose recommended for children). The fourth group was represented by rats that received 8 mg/kg ibuprofen (the standard dose recommended for children). The fifth group was represented by rats that were administered 20 mg/kg cefaclor (the standard dose recommended for children).

To create the groups, 15 nulliparous females weighing approximately 200 g were selected, and each of them was placed in a cage with a male of the same breed. Every 24 h, the vagina of the females was inspected by the veterinary technician to see if mating was achieved. After mating, that day was considered the first day of gestation. The gestational state was also confirmed by weight gain. On the sixth day of gestation, the control group was separated, and drug administration was started for the other 4 groups.

**Figure 1:**
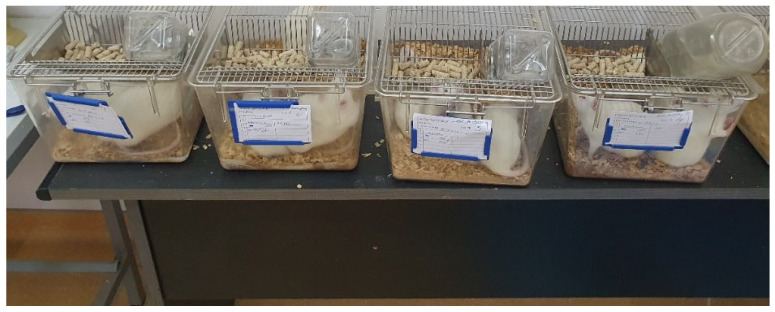
The groups of rats to which drugs were administered.

The cages were ventilated, with a 12 h light/dark cycle, and were maintained at a temperature of 25 ± 1 °C. The rats were fed with standard food—granulated combined fodder and water “ad libitum”. For each rat, a record was kept of the experiment to which it was subjected. The drugs were administered in a daily dose, orally, with a metal esophageal catheter, until the end of gestation. The animals were weighed daily to calculate the required dose. At the end of the gestation process, the date of birth was recorded for all pups. Pups were kept with their mothers until weaning (Figure 1).

To make up the final batches, 15 pups that had the highest weight from each batch were chosen. At the age of two months, rat pups were sacrificed according to current standards, after administration of an anesthetic overdose of 100 mg/mL Ketamidor 20 IU (0.2 mL) and 0,3 mL Xilazyn Bio 2%. The injection was performed intraperitoneally, slightly to the right of the abdominal white line.

All the phases related to the fertilization of the female rats, the administration of the drugs, the selection of the pups that were sacrificed, and the collection of the samples that were examined macroscopically by bright field microscopy (BFM) and PLM, which were carried out within the Biobase of the University of Medicine and Pharmacy of Craiova, Romania.

After slaughter, the maxilla and mandible of each pup were placed in 10% formalin for 48 h [11]. After 48 h, professional brushing and macroscopic examination of all dental arches (all maxillary and mandibular teeth of the 75 pups) were performed using the consultation kit, air spray, light source from the unit, and a magnifying glass with a diameter of 90 mm.

Following the macroscopic examination and identification of enamel defects, the samples underwent microscopic analysis using both BFM and PLM (cross-polarizer configuration). The microscopic examination was conducted with a Leica DM2500 microscope (Leica Microsystems, Wetzlar, Germany) in reflection mode. A 4× (N PLAN EPI 5×/0.10 POL) objective was employed, and for obtaining detailed images of the defects, a 10× (N PLAN EPI 10×/0.25 POL) objective was utilized. The system captured images using a 5MP CCD camera with auto-exposure software functionality enabled. The microscope’s light source was a tungsten-halogen lamp.

The visualization of the teeth surface was made in reflection mode, perpendicular to the contact area of the jaws. In order to obtain the acquisition for BFM, after the PLM image was acquired in cross polarizers’ configuration, the polarizer was taken out from the optical path of the microscope. In this way, for the same area visualized under a microscope, two images were acquired, one for BFM and one for PLM. The purpose of this procedure was to determine whether BFM could accurately localize the areas of teeth affected by dental developmental defects of enamel (DDE) with the same precision as PLM.

The brightness, contrast, and sharpness of the obtained photomicrographs were adjusted in order to increase the observable level of detail. All these operations were performed as a post-acquisition operation.

**Figure 2:**
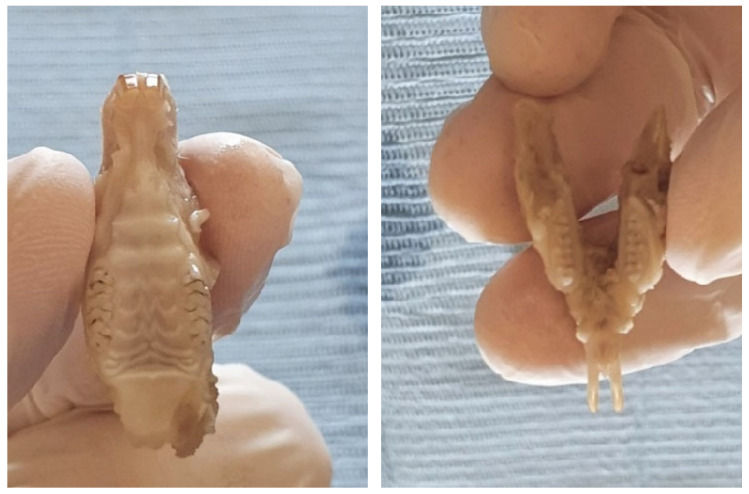
Images of maxillary (**left**) and mandibula (**right**) after preservation in formalin in order to prevent denaturation.

**Figure 3:**
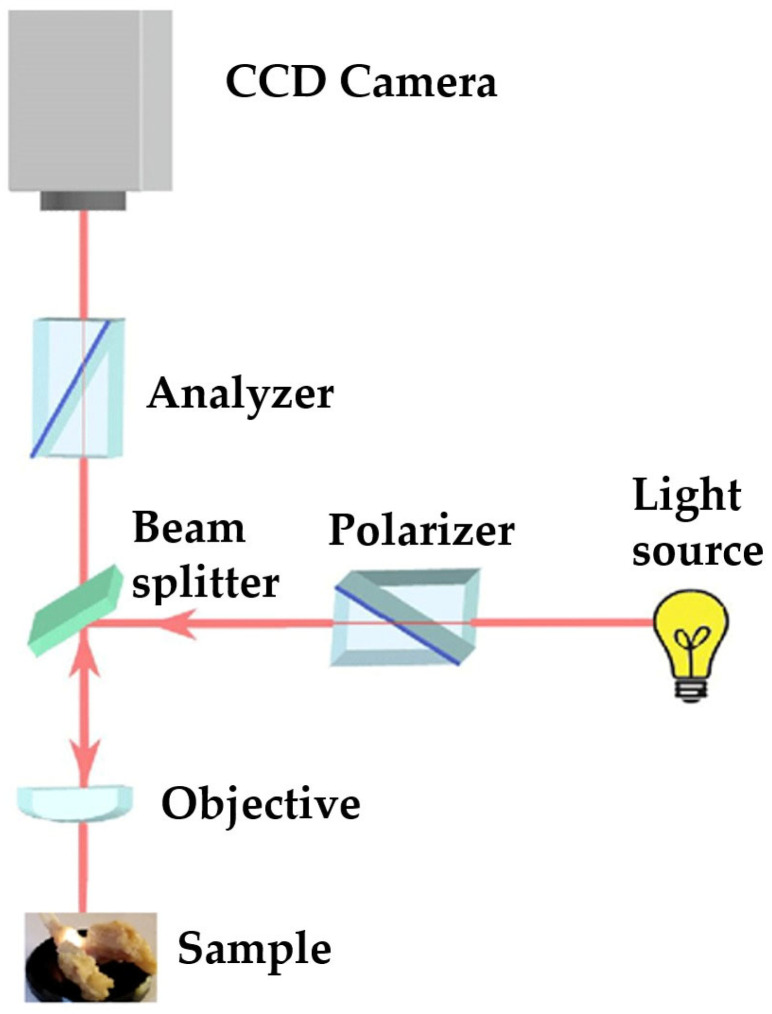
Optical scheme of polarized light microscopy.

Until the start of the microscopic study, the jaws were preserved in 10% formalin to prevent their denaturation (Figure 2). Before imaging, the samples were left in open air for several minutes to allow the liquid to evaporate from the examined surfaces. Wiping the samples with different tissues or paper was avoided, so as not to bring new particles to the tooth surfaces. For microscopic examination, the jaws were placed horizontally on the rotating microscope stand (Figure 3).

## 3. Results

Wistar rats have eight teeth in total—two central incisors and six molars in each jaw. Following a macroscopic analysis of the dental arches, no deviations from the typical anatomical features were found, including no dental agenesis or anomalies in the size or position of the teeth. DDE was defined macroscopically as enamel discontinuities that appeared punctiform or linear (hypoplasia) and opacities on the tooth surfaces (demineralization).

Not all structural defects could be highlighted by BFM; however, more enamel defects were seen using PLM. The number of DDEs captured by PLM is represented by the data that will be shown (Table 1).

**Figure 4:**
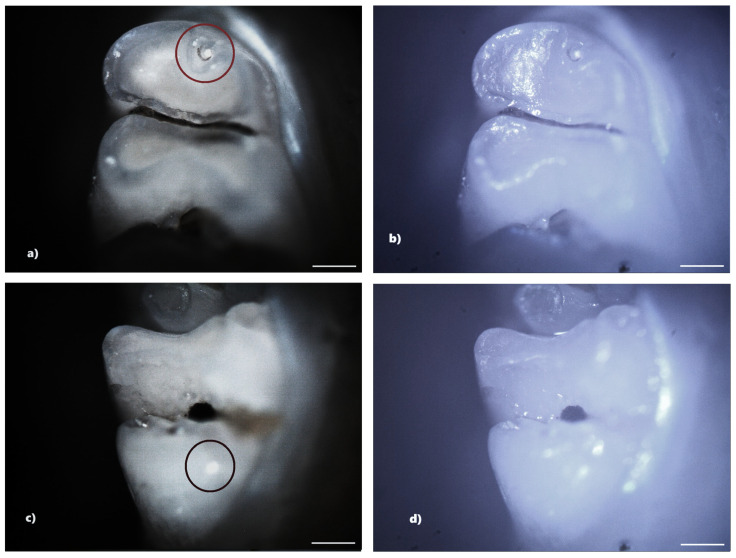
Demineralization on the occlusal surface of 2 molars (**a**,**c**—PLM image, cross polarizers, 4× objective; **b**,**d**—BFM image, 4× objective). Scale bar represents 500 μm.

From the control group, five rats (33.33%) showed reduced demineralization in extent. Of these, three rats showed demineralization in the incisors, and two rats showed demineralization in the molars. No rats from the control group showed hypoplasia. Within this lot, out of a total number of 240 teeth, 10 teeth (4.17%), 4 incisors, and 6 molars showed demineralization. For each of the 10 affected teeth, the lesion was localized on a single surface, resulting in a total of 10 tooth surfaces with DDE (Figure 4).

**Figure 5:**
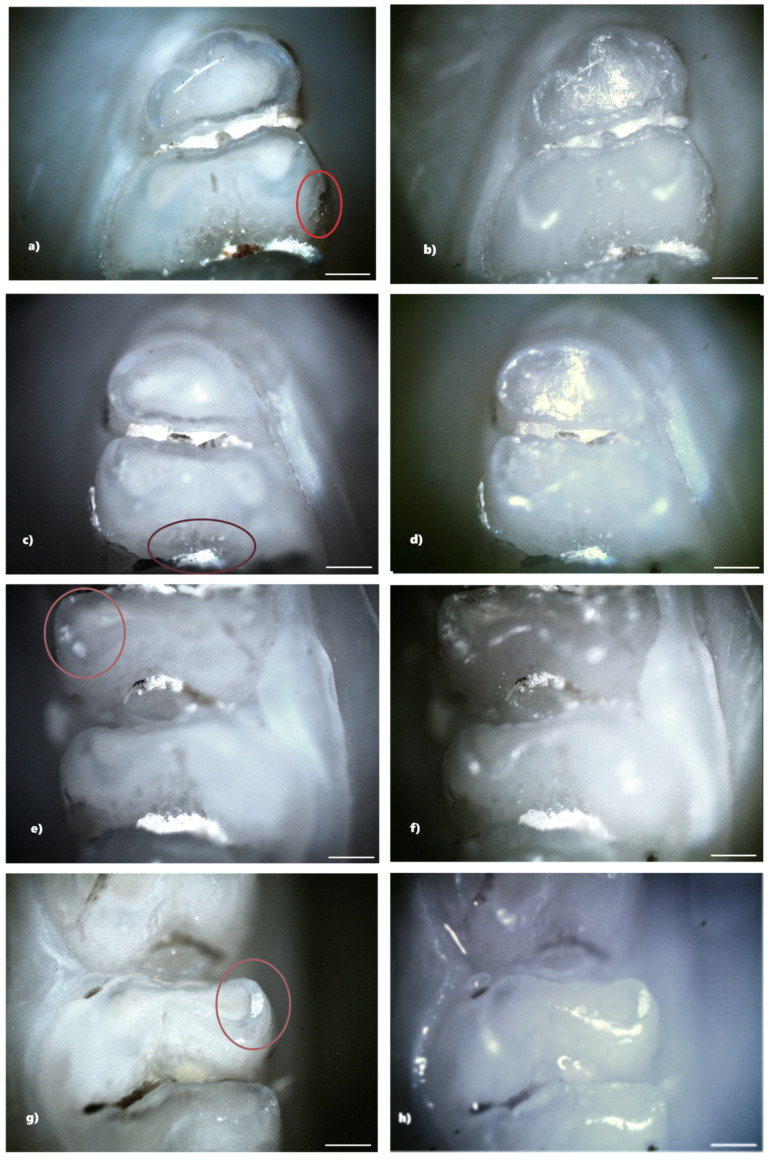
Hypoplasia on the palatal surface of a molar (**a**—PLM image, cross polarizers, 4× objective; **b**—BFM image, 4× objective) and on the distal surface of a molar (**c**—PLM image, cross polarizers, 4× objective; **d**—BFM image, 4× objective); demineralization on the occlusal surface of 2 molars (**e**,**g**—PLM image, cross polarizers, 4× objective; **f**,**h**—BFM image, 4× objective). Scale bar represents 500 μm.

In the standard dose amoxicillin group (50 mg/kg), 10 rats (66.67%) showed at least one clinical form of DDE. Of these, six rats showed hypoplasia, and three rats showed demineralization. One rat showed both clinical forms of DDE. Within this group, out of a total number of 240 teeth, 25 teeth (10.42%) showed at least one clinical form of DDE. Among them, 8 teeth (32%) were incisors and 17 teeth (68%) were molars, and as a clinical form of DDE, 16 teeth (64%) showed hypoplasia (five incisors and eleven molars) and 9 teeth (36%) showed demineralization (three incisors and six molars). Among the 25 teeth with DDE, 12 teeth (48%) presented lesions that were located on two surfaces, thus resulting in a total of 37 tooth surfaces with DDE (Figure 5).

**Figure 6:**
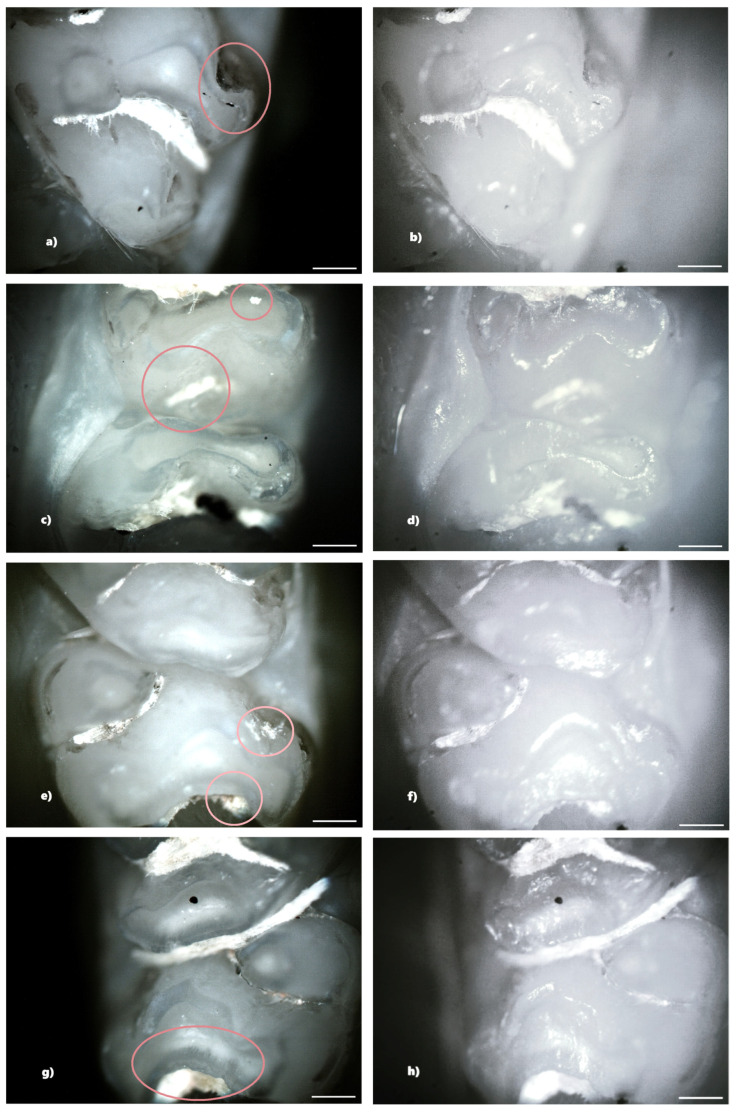
Hypoplasia on the palatal surface of a molar (**a**—PLM image, cross polarizers, 4× objective; **b**—BFM image, 4× objective) and on the distal surface of some molars (**e**,**g**—PLM, cross polarizers, 4× objective; **f**,**h**—BFM image, 4× objective); demineralization on the occlusal surface of a molar (**c**—PLM image, cross polarizers, 4× objective; **d**—BFM image, 4× objective) and on the buccal surface of a molar (**e**—PLM image, cross polarizers, 4× objective; **f**—BFM image, 4× objective). Scale bar represents 500 μm.

In the group receiving double-dose amoxicillin (100 mg/kg), 13 rats (86.67%) showed at least one clinical form of DDE. Of these, seven rats were affected by hypoplasia and five rats were affected by demineralization. One rat showed both clinical forms of DDE. Within this group, out of a total number of 240 teeth, 43 teeth (17.92%) had at least one lesion. Among them, 13 teeth (30.23%) were incisors and 30 teeth (69.77%) were molars, and as a clinical form of DDE, 25 teeth (58.14%) showed hypoplasia (5 incisors and 20 molars) and 18 teeth (41.86%) showed demineralization (8 incisors and 10 molars). Among the 43 teeth with DDE, 3 teeth showed DDE on two surfaces, resulting in 46 affected tooth surfaces (Figure 6).

**Figure 7:**
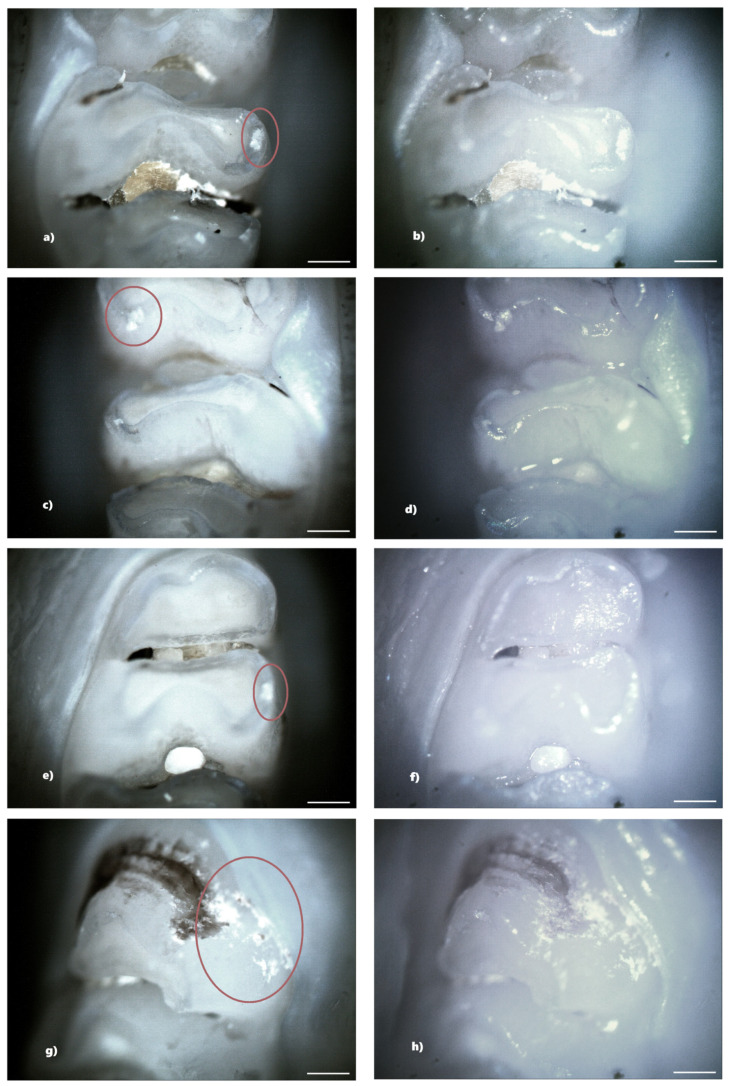
Demineralization on the occlusal surface of some molars (**a**,**c**,**e**—PLM image, cross polarizers, 4× objective; **b**,**d**,**f**—BFM image, 4× objective) and on the mesial surface of a molar (**g**—PLM image, cross polarizers, 4× objective; **h**—BFM image, 4× objective). Scale bar represents 500 μm.

In the standard dose ibuprofen group (8 mg/kg), 11 rats (73.33%) showed at least one clinical form of DDE. Of these, two rats showed hypoplasia and five rats showed demineralization. In addition, three rats showed both clinical forms of DDE. Within this group, from a total number of 240 teeth, 33 teeth (13.75%) presented at least one clinical form of DDE, respectively, 15 incisors (45.45%) and 18 molars (54.55%), and according to the clinical form of DDE, 7 teeth (21.21%) showed hypoplasia (molars) and 26 teeth (78.79%) showed demineralization (15 incisors and 11 molars). Each tooth with DDE had only one affected surface, resulting in a total of 33 affected tooth surfaces (Figure 7).

**Figure 8:**
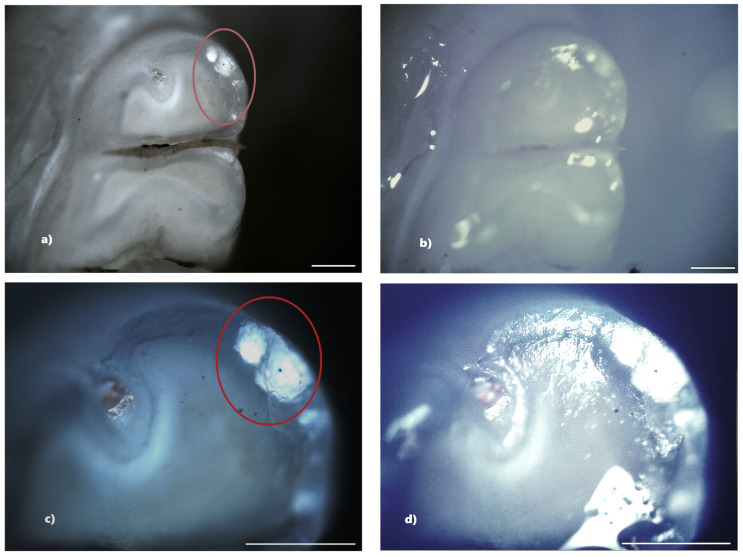

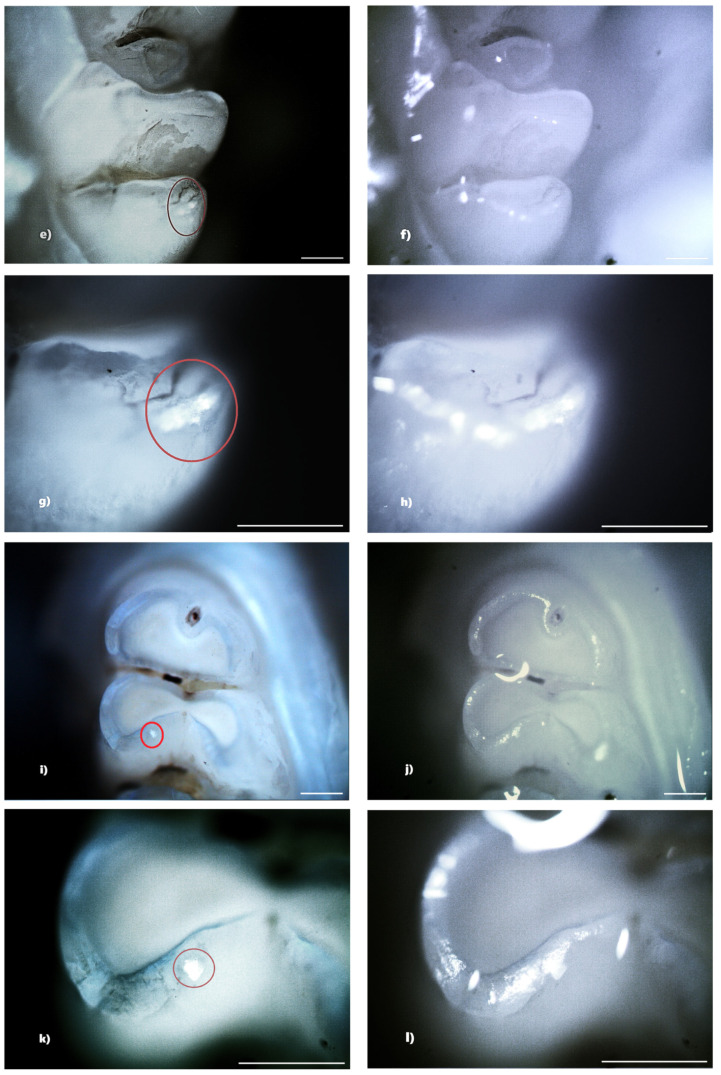
Demineralization on the occlusal surface of some molars (**a**,**e**,**i**—PLM image, cross polarizers, 4× objective; **c**,**g**,**k**—PLM image, cross polarizers, 10× objective; **b**,**f**,**j**—BFM image, 4× objective; **d**,**h**,**l**—BFM image, 10× objective). Scale bar represents 500 μm.

In the standard dose cefaclor group (20 mg/kg), 14 rats (93.33%) showed at least one clinical form of DDE. Of these, five rats showed demineralization and seven rats showed both clinical forms of DDE. Within this group, from a total number of 240 teeth, 45 teeth (18.75%) presented at least one type of DDE, respectively, 20 incisors (44.44%) and 25 molars (55.56%), and as a clinical form of DDE, 15 teeth (33.33%) showed hypoplasia (2 incisors and 13 molars) and 30 teeth (66.67%) showed demineralization (18 incisors and 12 molars). Among the 45 affected teeth, 10 teeth showed DDE on two surfaces, resulting in 55 affected tooth surfaces (Figure 8).

**Figure 9:**
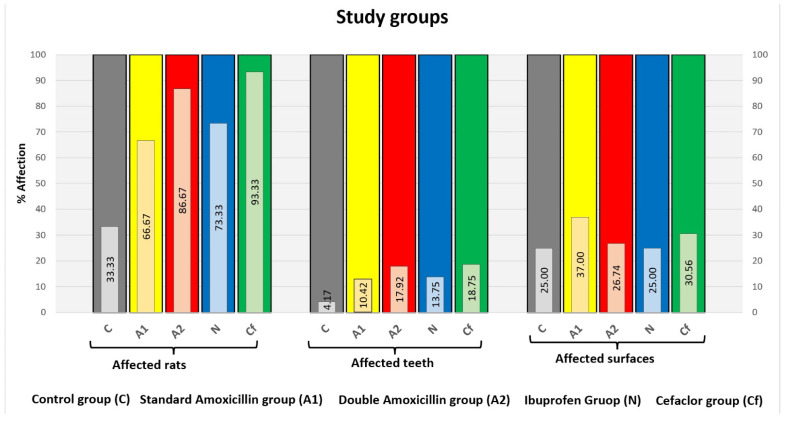
Centralizing graph of the 5 study groups.

Centralizing these data, it resulted that the group to which cefaclor was administered was the most affected (14 rats, respectively, 45 teeth with 55 affected surfaces), followed by the group to which amoxicillin was administered in a double dose (13 rats, respectively, 43 teeth with 46 affected surfaces), the group to which ibuprofen was administered (11 rats, respectively, 33 teeth with 33 affected surfaces), the group to which amoxicillin was administered in a standard dose (10 rats, respectively, 25 teeth with 37 affected surfaces) and the control group (5 rats, respectively, 10 teeth with 10 affected surfaces). It is clear that in the case of administration of cefaclor and amoxicillin in double dose and in standard dose, there were also teeth with more than one affected surface (Figure 9).

## 4. Discussion

The present study is a follow-up of the statistical study previously carried out and represents, in fact, an “in vivo” validation of the results obtained in the etiological study [10]. The choice of the drugs that were administered to reproduce in the laboratory the conditions that can generate DDE during the period of odontogenesis was made according to the results of the previously performed etiological study.

Similar studies in rats have also been conducted, but generally, fewer drugs or smaller numbers of rats were used. Thus, in 2019, Kameli used standard and double-dose amoxicillin and standard-dose tetracycline; in 2018, Serna Muñoz used amoxicillin, amoxicillin with clavulanic acid, erythromycin, paracetamol, ibuprofen, and celecoxib; in 2015, de Souza used standard and five times higher doses of amoxicillin; in 2014, Gottberg used standard and double-dose amoxicillin and standard-dose tetracycline; and in 2013, Sahlberg used amoxicillin [11,12,13,14,15].

The identification of DDE was carried out in the present study by macroscopic examination, BFM and PLM, effectuated in reflection mode. The results indicated that PLM facilitates the identification and delineation of DDE defects much more accurately than BFM because of the lack of shiny surfaces. The photomicrographs obtained using PLM demonstrated superior image contrast and quality compared to BFM.

The configuration used for the analysis of the jaws was that of crossed polarizers, thus ensuring a higher contrast in observing areas affected by DDE, areas that scatter and strongly depolarize the light. In the case of crossed polarizers, areas that change the polarization state of the reflected light will appear bright, and those that do not present such properties will appear dark.

Compared to BFM, PLM allows easier identification of DDE, as well as their delimitation. Furthermore, the suppression of light reflected from the tooth surface (enamel-air interface) enables the visualization of all sample details, avoiding the presence of shiny areas characteristic of BFM. DDE-affected regions are readily identifiable by their distinct white scattered light fingerprint, which contrasts with the adjacent areas. We consider that the depolarization of light occurring at the interface between enamel and dentin, as well as within DDE regions, is the primary factor contributing to the increased brightness observed in certain areas of the teeth. Despite the fact that enamel is birefringent and can induce multiple scattering events, leading to changes in the polarization state of the incident light, the surface under the email is seen as clear, without interference patterns [16].

The crystalline structure of enamel consists of hydroxyapatite crystallites with varying orientations. The current findings show the effectiveness of PLM in identifying DDE, a conclusion that is supported by other research groups [16].

Gottberg used optical microscopy to identify DDE. In the present study, by macroscopic examination of the teeth, no variations of the normal anatomical features were identified. Similar results were reported by Gottberg in a similar study [11]. The results obtained in the laboratory, regarding the effects of drugs administered during odontogenesis, were much more conclusive than in the etiological study; thus, demineralization and even hypoplasia were identified in a greater proportion. DDE present in the control group (in a reduced proportion) should be noted, suggesting a number of other factors involved in the development of DDE.

Administration of standard and double-dose amoxicillin to pregnant female rats determined the presence of hypoplasia and demineralization in their offspring. Amoxicillin is a semi-synthetic penicillin of the Beta-lactam family with a broad spectrum and with bactericidal activity against gram-positive and gram-negative bacteria [11]. Amoxicillin is prescribed as the antibiotic of choice for the treatment of respiratory, gastrointestinal, genital, skin, and neurological infections [17]. In children, it is the most commonly prescribed antibiotic [18]. In the dental field, it is administered against microorganisms associated with dental infections, being at the same time the antibiotic of choice in the prophylaxis of bacterial endocarditis [19,20]. The most common side effects of amoxicillin are hypersensitivity reactions and gastrointestinal disorders [17].

Although in the specialized literature amoxicillin is frequently associated with demineralization, in the present study, the groups to which this antibiotic was administered developed a higher percentage of hypoplasia. In a review on the etiology of DDE in temporary teeth, amoxicillin was mentioned as a risk factor for hypoplasia [21].

A series of studies have proven that amoxicillin affects the thickness of the ameloblastic layer and implicitly the thickness of the enamel, hypoplasia being quantitative defects [12,14,15]. De Souza observed vacuole-like structures in the ameloblastic layer of amoxicillin-treated rats and concluded that these structures arose because of the antibiotic’s interference with the transmitter’s environment, leading to reduced protein secretion and transmission [14]. Reduction in enamel thickness was also reported by Sahlberg, who, in an “in vitro” study, exposed the erupted teeth of rats to amoxicillin. His explanation was that the antibiotic prevented ameloblastic differentiation [15]. The results of the present study, regarding the demineralization that occurred as a result of amoxicillin administration, were also confirmed by other “in vitro” studies [11,22].

Following the histological examination, Gottberg identified demineralization in the enamel of all examined samples from the 100 mg/kg amoxicillin group. Only half of the examined samples from the 50 mg/kg amoxicillin group showed demineralization. These results coincided with the present study’s results, which demonstrate that the drug’s effects on teeth are directly proportional to the administered dose [15].

Incorporation of amoxicillin into developing teeth is favored by the fat-solubility of the antibiotic (superior to other penicillins) to the large number of blood vessels found in the intermediate layer of the molars intercuspid regions and by the low affinity of this drug due to plasma proteins [17]. The mechanism by which some antibiotics affect tooth enamel has been attributed to the fact that these drugs alter protein synthesis [11]. Recently, Sahlberg demonstrated that amoxicillin decreases the level of the MMP 20 metalloproteinase, which has an important role in the degradation and removal of enamel proteins [15]. Gottberg and Laisi concluded that the antibiotic alters the normal temporal sequence of tooth enamel development [11,22]. The widespread administration of amoxicillin to both pregnant women and children, in the absence of clear evidence of the antibiotic effect on the developing dentition, suggests the need to determine whether there is a connection between the antibiotic and DDE [11].

Ibuprofen is a drug that belongs to the group of non-steroidal anti-inflammatory drugs, being used in many countries for the treatment of inflammation and conditions manifested by pain and fever [23]. Ibuprofen is part of several medicines: Nurofen (tablets for adults, syrup for children), Brufen (tablets), Ibufen (tablets), and Ibalgin (tablets). All of these can be bought over the counter in pharmacies in Romania. The use of ibuprofen in pediatrics has been reviewed, with results reporting that the drug can be administered safely and is effective against acute pain and fever [23]. In general, for infants and children up to the age of 12, the most used form of presentation of ibuprofen is Nurofen in the form of syrup. It has the effect of relieving fever and pain, being recommended in fever after vaccination or in various other conditions accompanied by fever and pain (pharyngitis, tonsillitis, tooth eruption accidents or complicated caries, inflammatory diseases of the ear, minor traumas). In dentistry, ibuprofen is used to reduce pain and inflammation, especially in teeth diagnosed with acute pulpitis or acute apical periodontitis [24]. A number of studies have shown that long-term use presents a relatively small risk for hepato-renal or gastrointestinal adverse effects [23].

In the specialized literature, there is only one study in which the involvement of ibuprofen in the development of DDE was investigated [13]. The study was carried out on mice, which were administered several drugs, including ibuprofen, for 30 days from the 21st day of life. Following scanning electron microscopy and energy dispersive X-ray analysis, Serna Muñoz found that ibuprofen did not influence the level of calcium and phosphorus, having no role in the occurrence of demineralization [13].

These results are in contradiction with the present study’s results, in which 33 teeth with DDE were identified, most of them in the form of demineralization. However, in the present study, ibuprofen was administered prenatally at a daily dose of 8 mg/kg, while Serna Muñoz administered it postnatally at a dose of 2.5 mg/day. In addition, the methods of drug administration were different, as were the teeth chosen for examination. Thus, Serna Muñoz administered ibuprofen in strawberry gelatin, while a metal esophageal catheter was used in the present study. Further, in the present study, all teeth were examined, while Serna Muñoz examined only third molars.

Serna Muñoz explained the possible mechanism by which ibuprofen can affect amelogenesis, producing demineralization [13]. According to her, NSAIDs exert their anti-inflammatory effect by inhibiting cyclooxygenase (COX) and prostaglandin synthesis. Cyclooxygenases are enzymes that catalyze the formation of prostaglandins from arachidonic acid by mediating physiological processes, including inflammation. At least two different enzymes are involved in this mechanism: constitutive COX1 (cyclooxygenase 1) and inductive COX2 (cyclooxygenase 2), closer to inflammatory processes. COX2 expression is transiently increased in response to inflammatory stimuli. Induction of COX2 results in rapid augmentation of intracellular levels of nitric oxide, cytokines, and intracellular calcium [22]. It is possible that the mineralization phase of enamel requires inflammation mediators because ameloblasts require a large influx of ions during crystal formation. Serna Muñoz proved that COX2 is present in the enamel organ. Inhibition of COX2 activity could produce a reduction in prostacyclin, which could decrease the blood flow in the ameloblasts area. During the maturation phase of amelogenesis, the rapid diffusion of nutrients at the level of the ameloblastic layer is necessary, as well as the rapid incorporation of ions, for the correct development of crystals [25]. Prostacyclin reduction is also likely to affect the buffering capacity of the local tissue involved in maintaining pH homeostasis at the level of the enamel mineralization matrix, which is essential for healthy enamel [24]. Therefore, COX2 is involved in the maturation phase of the enamel organ and its inhibition, resulting in the disruption of amelogenesis and the appearance of demineralization [13].

Through immunohistochemistry techniques, Serna Muñoz highlighted the fact that ibuprofen produced an average decrease in the amount of immunoreactive COX2, which was considered insignificant. The author also reported that another nonsteroidal anti-inflammatory drug, celecoxib, did not reduce the amount of COX2, a contradictory result since celecoxib is known as a selective inhibitor of COX2 [13]. In 2008, Vardar-Sengul explained these mechanisms by the fact that celecoxib could reduce COX2 activity and not the expression or amount of the enzyme [25]. Thus, it can be considered that the demineralization highlighted in the present study, in the group to which ibuprofen was administered, may be based on the inhibition of COX2 activity.

Cefaclor is an antibiotic that is part of the 2nd generation of the Cephalosporin family, being used more and more frequently in medical practice to treat a wide variety of infections [26]. Its widespread use is due to the fact that it has low toxicity and allergenic effects, being effective against a wide spectrum of conditions [27,28]. Cefaclor has antibacterial activity against gram-positive and gram-negative aerobic microorganisms [29]. Cefaclor is recommended in the treatment of pneumonia, acute bronchitis, acute sinusitis, pharyngitis, tonsillitis, otitis media, skin and soft tissue infections, and urinary tract infections [28]. Cefaclor is available as capsules for adults and oral suspension for children. In dentistry, the antibiotic is used systemically as a treatment for abscesses generated by dental infections, and, in endodontics, being a component of the triple antibiotic paste, applied intracanally for disinfectant and pulp regeneration actions [30]. Among the reported side effects are pruritus, rashes, indigestion, and diarrhea [29,31].

According to the present study, among the drugs used, cefaclor induced the highest number of DDE in the form of demineralization, most frequently, and hypoplasia. Other studies have also associated the administration of cefaclor in children with the development of DDE [8,9,32]. Tariq’s 2014 study was based on the examination of children and the filling of a questionnaire by parents [31]. Tapias conducted a retrospective study in 2001 in which he analyzed the association between various risk factors of DDE on the first permanent molar [9]. Thus, he followed the medical history of 48 children during the first 5 years of life and found a statistically significant association between the administration of cefaclor at the age of 4 and DDE. In a case report, Simratvir reported, in 2011, the existence of hypoplasia in all erupted and unerupted permanent teeth of a 9-year-old girl [8]. One of the 6-year-old molars was also affected by demineralization. Following the patient’s medical history, Simratvir concluded that the only factors possibly involved in the development of DDE were the frequent presence in the first 4 years of life of upper respiratory tract conditions and the medication used as treatment. The parents gave her cefaclor and paracetamol for almost 4 years because of the recurrence of the respiratory infection.

In another study, de Sousa used PLM to describe fluorotic lesions he induced on rat incisors. Adult experimental animals were given drinking water containing 45 mg F/L of fluoride, while distilled water was given to the control group. The authors concluded that white bands of fluorotic rat enamel are not subsurface lesions; rather, they are hypomineralized superficial regions [33].

In the specialized literature, however, there are also studies that address the association between antibiotics and DDE, with opposite results compared to the previously presented studies. Thus, in a study carried out in 2009 on 147 children with an average age of 10.7 years, the association of several antibiotics, including cefaclor, with the development of demineralization was investigated. A direct relationship between the use of cefaclor and the presence of demineralization was not identified [22].

To our knowledge, there are no explanations in the specialized literature of how cefaclor intervenes in odontogenesis and causes the development of DDE. The differences between the results reported by the studies addressing this topic can be attributed to the different drugs and doses used, the duration and routes of administration, the different moments of pup slaughtering, as well as the teeth chosen for the study [11].

The present study demonstrates the importance of re-evaluating the adverse effects of the administration of some drugs during pregnancy and in the first years of the child’s life and possibly limiting the prescription of these drugs to pregnant women and children in cases where it is strictly necessary. In addition, the replacement of these drugs with others that are not associated with DDE should be considered.

It is useful to educate pregnant women and mothers about the prevention methods of DDE and the risk that these drugs present for the development of DDE. Although in the previous statistical study, the drugs (amoxicillin, ibuprofen, cefaclor) highlighted as having a role in the development of DDE were administered postnatally, and in the present study they were administered prenatally, it is believed that in both cases, they influenced odontogenesis [10]. In studies from the fields of fuel and particuology, other detection methods, such as optical microscopy, have proven to be effective. Thus, under an optical microscope, the micrograph, particle size distribution, and dispersion of an innovative emulsified waste fried oil collector of the oil-in-water type were examined. It was produced via ultrasonic emulsification and transesterification, and it may be utilized as a substitute collector to increase low-rank coal flotation performance while using less collector dose [34,35]. No rat studies were found in the specialty literature that used four drugs to produce DDE simultaneously under the same conditions.

## 5. Conclusions

The present study was carried out “in vivo” on Wistar rats and had the objective of highlighting the role of some drugs (amoxicillin, ibuprofen, cefaclor) administered during pregnancy in the development of DDE. To carry out the study, five groups of rat pups were formed: the control group, the group that was administered standard dose amoxicillin, the group that was administered double dose amoxicillin, the group that was administered standard dose ibuprofen and the group that was administered standard dose cefaclor.

Examination of rat pups’ teeth was done macroscopically and by BFM and PLM. In the control group, DDE was identified in a reduced number, resulting in the fact that there are other factors involved, besides the drugs administered, in the development of DDE. The highest number of DDE was recorded in the cefaclor group, followed by the double-dose amoxicillin group, the ibuprofen group, and the standard-dose amoxicillin group. DDE in the form of demineralization was more frequently recorded in the cefaclor and ibuprofen groups. DDE in the form of hypoplasia was more frequently recorded in the double-dose and standard-dose amoxicillin groups.

## Figures and Tables

**Table 1:** DDE centralization (demineralization, hypoplasia) in the 5 groups of rats, according to the number of affected teeth.

	Control		Amoxicilin		Amoxicilin		Ibuprofen		Cefaclor	
			50 mg/kg		100 mg/kg		8 mg/kg		20 mg/kg	
	Nr	%	Nr	%	Nr	%	Nr	%	Nr	%
**DDE teeth**	10	100.00	25	100.00	43	17.92	33	100.00	45	100.00
**Incisors**	4	40.00	8	32.00	13	30.23	15	45.45	20	44.44
**Molars**	6	60.00	17	68.00	30	69.77	18	54.55	25	55.56
**Demineralization**	10	100.00	9	36.00	18	41.86	26	78.79	30	66.67
**Incisors**	4	40.00	3	12.00	8	18.60	15	45.45	18	40.00
**Molars**	6	60.00	6	24.00	10	23.26	11	33.33	12	26.67
**Hypoplasia**	0	0.00	16	64.00	25	58.14	7	21.21	15	33.33
**Incisors**	0	0.00	5	20.00	5	11.63	0	0.00%	2	4.44
**Molars**	0	0.00	11	44.00	20	46.51	7	21.21	13	28.89

## Data Availability

The data presented in this study are available on request from the corresponding author. The data are not publicly available due to privacy.
